# High-Throughput Sequencing and Linkage Mapping of a Clownfish Genome Provide Insights on the Distribution of Molecular Players Involved in Sex Change

**DOI:** 10.1038/s41598-018-22282-0

**Published:** 2018-03-06

**Authors:** Laura Casas, Pablo Saenz-Agudelo, Xabier Irigoien

**Affiliations:** 1 King Abdullah University of Science and Technology (KAUST), Division of Biological and Environmental Science & Engineering, Red Sea Research Center, Thuwal, 23955-6900 Saudi Arabia; 2 Present Address: Institute of Marine Research (IIM-CSIC), 36208 Vigo, Spain; 3 Present Address: Instituto de Ciencias Ambientales y Evolutivas, Universidad Austral de Chile, Valdivia, 5090000 Chile; 4 AZTI - Marine Research, Herrera Kaia, Portualdea z/g –, 20110 Pasaia, Gipuzkoa Spain; 5 IKERBASQUE, Basque Foundation for Science, Bilbao, Spain

## Abstract

Clownfishes are an excellent model system for investigating the genetic mechanism governing hermaphroditism and socially-controlled sex change in their natural environment because they are broadly distributed and strongly site-attached. Genomic tools, such as genetic linkage maps, allow fine-mapping of loci involved in molecular pathways underlying these reproductive processes. In this study, a high-density genetic map of *Amphiprion bicinctus* was constructed with 3146 RAD markers in a full-sib family organized in 24 robust linkage groups which correspond to the haploid chromosome number of the species. The length of the map was 4294.71 cM, with an average marker interval of 1.38 cM. The clownfish linkage map showed various levels of conserved synteny and collinearity with the genomes of Asian and European seabass, Nile tilapia and stickleback. The map provided a platform to investigate the genomic position of genes with differential expression during sex change in *A. bicinctus*. This study aims to bridge the gap of genome-scale information for this iconic group of species to facilitate the study of the main gene regulatory networks governing social sex change and gonadal restructuring in protandrous hermaphrodites.

## Introduction

Teleost fishes constitute the most diverse and species-rich clade among vertebrates, comprising over half of all known species. They display the largest array of sex-determining systems among animals, resulting in a large number of reproductive strategies, a key factor in explaining their success during evolution (see^1–3^ for reviews). Among these, sequential hermaphroditism is a unique strategy^4,5^ displayed across seven orders of teleosts, mainly in the coral reef environment. It has been proposed that sequential hermaphroditism in this environment improves adaptation; increases survival rates and enhances reproduction^6,7^.

Despite recent advances in next generation sequencing technologies (NGS), genome-scale resources are only available for a handful of fish species. High-density genetic linkage maps are essential in facilitating high quality *de novo* genome assemblies and in comparative genomics. The advent of NGS has made it possible to discover thousands of SNPs dispersed throughout the genome in a single procedure, even in non-model species. To date, high-density linkage maps using genotyping by sequencing approaches have been constructed in over 20 fish species such as the Midas cichlid^8^, Atlantic halibut^9^, Nile tilapia^10^ and the platyfish^11^, among others. For hermaphrodite species, high resolution maps have been developed in two protogynous (orange-spotted^12^; common pandora)^13^ and a protandrous hermaphrodite (Asian seabass)^14^.

However, the great majority of non-model fish species lack genome-scale data and this is especially true for non-model reef fish species, including clownfishes. The recently published transcriptome of the Red Sea clownfish *Amphiprion bicinctus*^15^ constitutes the first genome-scale resource for this group of iconic species that had virtually no genome resources before. This deficiency ballasts our capacity to elucidate the molecular pathways underlying reproductive processes in hermaphrodites, mutualism or evolution and adaption in this species.

Clownfishes (subfamily Amphiprioninae) are extensively distributed in tropical waters, where they inhabit shallow depths across the Red Sea, the Indian and the western Pacific Oceans^16^. They live in social assemblages as pairs or social groups consisting of a dominant female, always the largest in size, surrounded by a male and a variable number of juveniles of smaller size^17,18^. This group of fishes displays a protandrous monogamous mating system and a strong social hierarchy based on size that functions as strict queues for breeding^19–24^. If the dominant female of a social assemblage dies, all subordinates seize the opportunity to ascend in rank and grow, allowing the formation of a new breeding pair without the need for dangerous travel across the reef^25^. Sex change in clownfish is not size dependent and seems to be controlled socially^25–27^ but the genetic mechanism underlying this process remains largely unknown^3^.

*Amphiprion* species constitute a powerful system for investigating the genetic mechanism governing hermaphroditism and socially-controlled sex change in their natural environment since they are strongly site-attached. They are obligate symbionts of sea anemones and rarely stray farther than a few meters away from these microhabitats during their entire adult life^28,29^. This enables finding a given individual at the exact same location over time. Moreover, sex change can be triggered by simply removing the female dominant member in any given social assemblage, allowing a precise monitoring of the process in the field. Thus, they represent an ideal model to monitor and study sex change and mutualism in their natural environment.

Clownfishes in tropical regions typically have a year-round non-seasonal spawning pattern with strong lunar, or semi-lunar, periodicity in the timing of spawning’s^30,31^. They attach 500 to 800 capsule-shaped eggs in single-layer circular clutches to the benthos in semi-cryptic habitats within the periphery of their host anemone^32^. The parents tend these clutches of eggs until the embryos hatch about one week after fertilization, with well-developed skeletal, organ and sensory systems^33^.

Here, we aim to bridge the gap of genome-scale information for this iconic species to facilitate the study of the main gene regulatory networks involved in the sex change process. Capitalizing on the power of massively parallel sequencing to reveal genetic polymorphisms and rapidly characterize the genomes of non-model species, we present the first linkage map in the Red Sea endemic species of clownfish *A. bicinctus*. We used restriction-site associated DNA sequencing (RADseq), which allows the *de novo* discovery and simultaneous scoring of hundreds to thousands of single nucleotide polymorphism (SNP) markers from a single sequencing run for dozens of individuals, to generate the map. The main aim of the study was to construct a high-density linkage map of the *Amphiprion bicinctus* genome using SNP markers derived from a RAD-Seq analysis to provide a backbone for further genetic studies. An additional goal, building on this linkage map, was to explore the location of loci encoding genes potentially involved in sex change in this hermaphrodite species using the existing transcriptome.

## Results

### RAD data analysis

After demultiplexing, trimming and quality filtering a total of 497,598,696 high quality paired-end reads were retained. The average number of reads per sample ranged from 895,338 to 13,647,439 (average ~5.2 million reads per sample, SD 2.5 M) (Supplementary Table S1). In total 93% of the reads were used to build Stacks. The number of unique Stacks per sample ranged from 68,042 to 404,278 (average = 323,831, SD = 70,726), and the average depth of coverage per sample ranged from 6× to 38× (average = 16×, SD = 6.4). A catalog containing 283,946 loci was built from both male and female sequence data and all offspring were matched against this catalog. The percentage of Stacks per offspring that matched the catalog ranged from 80 to 92%. The number of polymorphic loci per offspring that matched the catalog ranged from 1057 to 5487 (average = 4,658, SD = 683). Finally, from the loci in the catalog, 56,910 were polymorphic. From these, 6056 loci were present in at least 80% of the progeny. A total of 2910 loci exhibited significant segregation distortion after applying a chi-square test (p < 0.05) and were discarded, resulting in a final dataset of 3146 polymorphic loci that was used in the subsequent linkage analysis.

Paired-end RAD-Seq results in sequence from both ends of the randomly-sheared genomic fragments which are anchored at the *SphI* cleavage sites. The resulting dataset includes the final RAD loci corresponding to the 95 bp immediately adjacent to the *SphI* cleavage site and an associated RAD contig resulting from the assembly of the associated paired-end read. The length distribution of RAD contigs produced, with an average length = 485.05 bp; median length = 542 bp, is shown in Supplementary Fig. S1.

### Clownfish map construction

**Figure 1:**
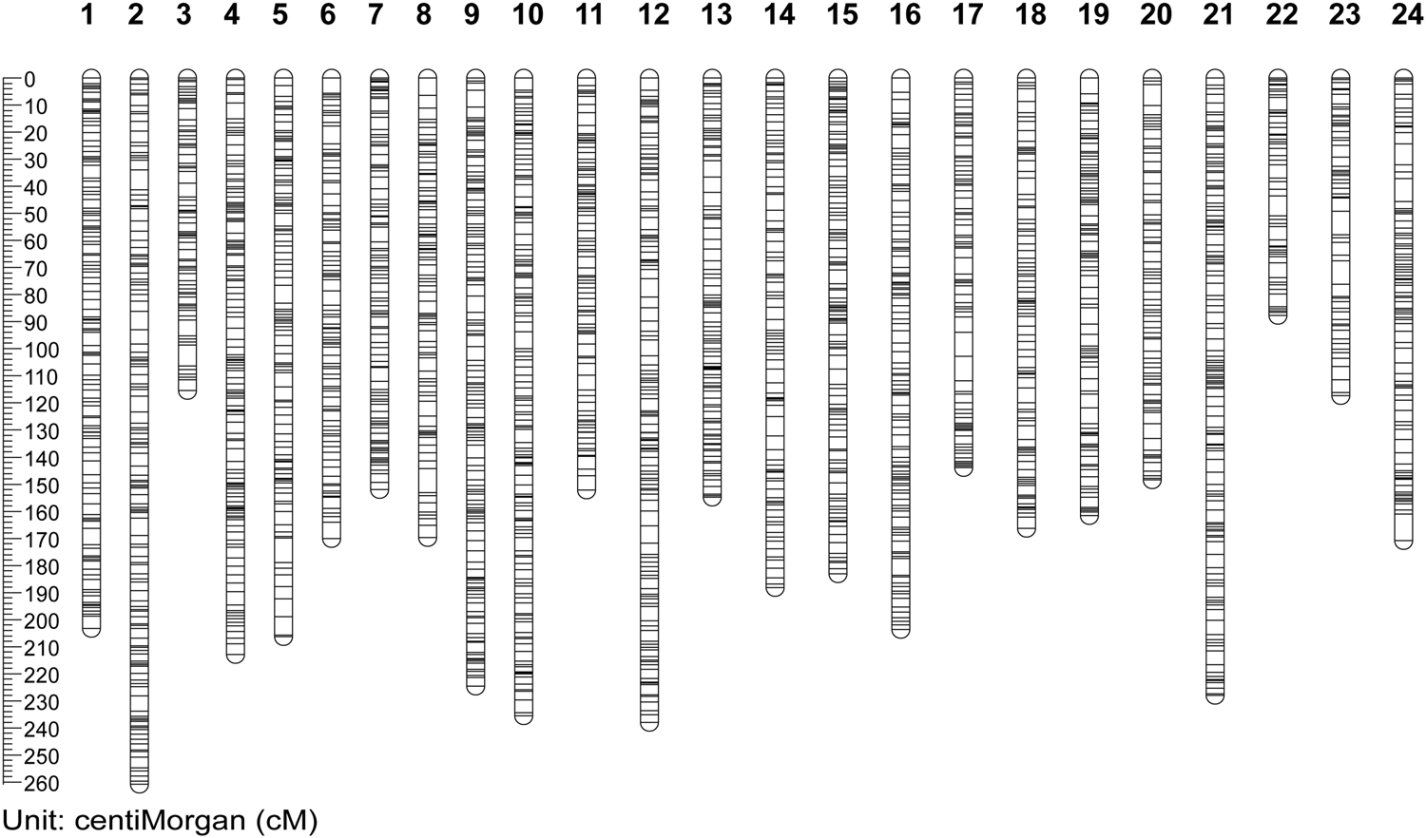
Linkage map of the Red Sea clownfish *Amphiprion bicinctus* produced from 3146 RAD loci distributed in 24 linkage groups. Map distances were calculated using the Kosambi function.

The 3146 informative RAD loci comprised bi-allelic markers heterozygous in both parental individuals with the following segregation patterns: ab/ab 1896, ab/ac 1114, and ab/cd 136. For the linkage mapping, a LOD score of 14 was selected resolving 24 linkage groups, designated as abLG1-abLG24 (Fig. 1). The number of linkage groups yielded corresponded to the haploid chromosome number of the Red Sea clownfish (see Methods for details). The constructed abLGs, contained from 65 to 179 RAD loci spanning 87.79 to 260.81 cM in length, with a mean distance between markers of 1.10–1.71 cM (Fig. 1 and Table 1). The total length of the map was 4294.71 cM, with an average inter-locus distance of 1.38 cM. The largest gap in the map was 9.82 cM in abLG24, followed by 9.18 cM in abLG17 (Supplementary Table S2).

**Table 1:** Summary of the Red Sea clownfish map.

Clownfish abLG	Size (cM)	Number RAD Loci	Mean distance between markers (cM)
1	203.2357744	147	1.392025852
2	260.8122798	176	1.490355885
3	115.5290105	88	1.327919661
4	212.7948591	169	1.266636066
5	206.266411	149	1.393691966
6	170.0938091	116	1.479076601
7	151.9575601	139	1.10114174
8	169.6410226	118	1.44992327
9	224.6401694	173	1.306047496
10	235.5013328	167	1.418682728
11	152.1028933	112	1.370296336
12	237.9905629	156	1.535422987
13	154.7149275	124	1.257844939
14	188.8340065	113	1.686017915
15	183.0451833	155	1.188605087
16	203.6771745	146	1.404670169
17	143.7544374	110	1.318848049
18	166.2292002	124	1.351456912
19	161.4636246	137	1.187232534
20	148.3704631	88	1.705407621
21	228.0358364	179	1.281100204
22	87.79384775	65	1.384601096
23	117.3821781	79	1.504899719
24	170.8397613	116	1.485563142

### Comparative genomic analysis

To validate the constructed linkage map, the genomic architecture of the Red Sea clownfish was compared to that of other teleosts by identifying the most similar homologous regions of clownfish RAD loci present in the genomes of Asian and European seabass, Nile tilapia and stickleback (Supplementary Table S3).

**Figure 2:**
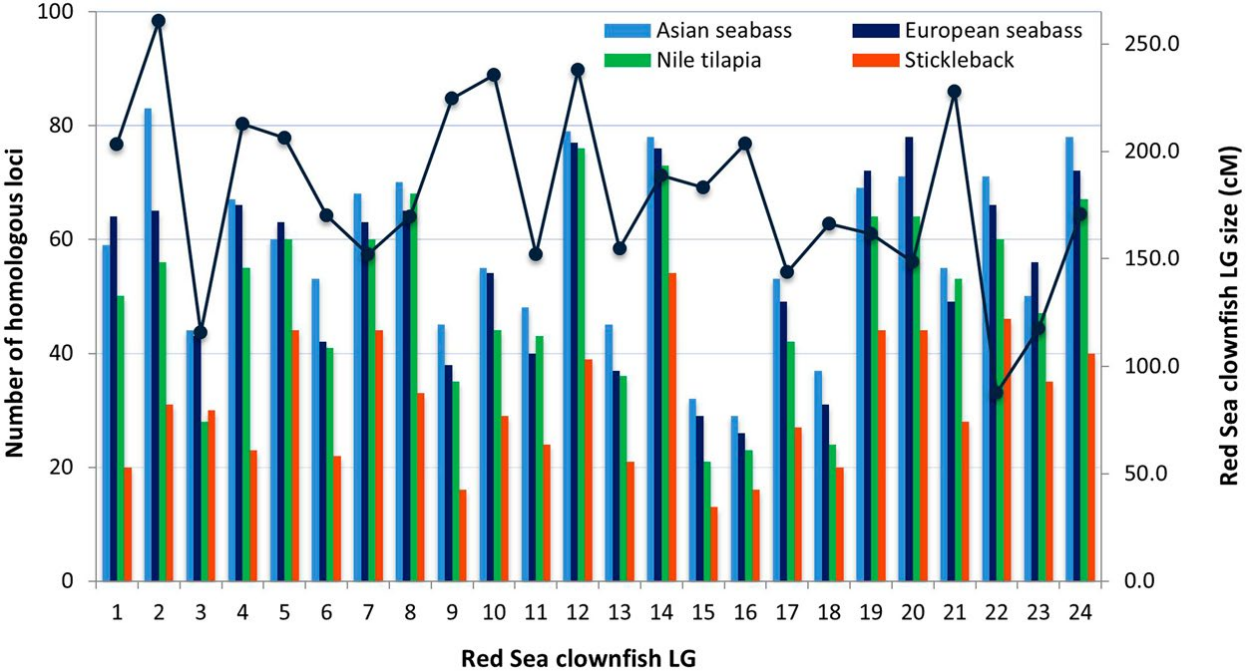
Chromosome-level comparative analysis of homologous loci for the Red Sea clownfish linkage groups (abLGs) with Asian seabass (light blue), European seabass (navy blue), Nile tilapia (green) and stickleback (orange). The bars indicate the number of homologous loci found in each of the four reference species and the line indicates the size of the clownfish linkage group (cM).

In addition, those RAD loci that failed to match at least one of these genomes were aligned to the recently published reference clownfish transcriptome assembly. This strategy provided anchors for positioning RAD loci that did not directly match any of the four reference teleost genomes. By doing this we were able to resolve the positioning of 313, 293, 321 and 294 additional transcriptome-anchored RAD markers in the genomes of Asian and European seabass, Nile tilapia and stickleback, respectively (Supplementary Table S4). The chromosome-level comparative analysis of loci mapped by both strategies revealed that 2038 (64.78%) of the total RAD loci on the map showed significant homology in at least one of the four reference genomes (Supplementary Tables S3 and S4). Significant macrosynteny was observed between *A. bicinctus* and all four species, with a variable number of homologous loci detected in each of them, ranging from 1399 (44.47% of all loci; Asian seabass), 1321 (41.99%; European seabass), 1190 (37.83%; Nile tilapia) and 743 (23.62%; stickleback) (Fig. 2, Supplementary Tables S3 and S4).

**Figure 3:**
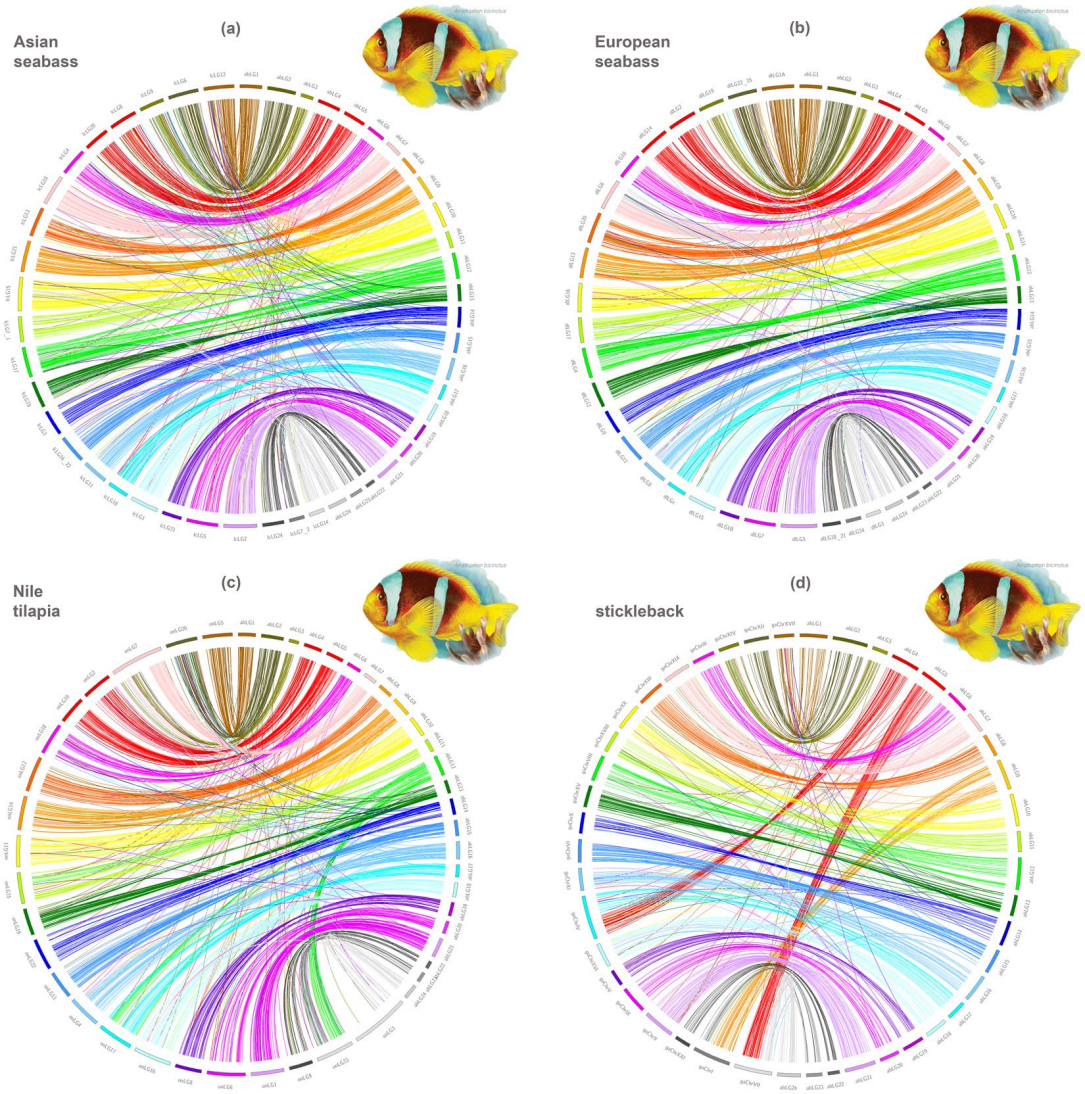
Genomic synteny between the Red Sea clownfish linkage groups and (**a**) Asian seabass, (**b**) European seabass, (**c**) Nile tilapia and (**d**) stickleback genomes, represented by Circos software.

Detailed analysis of the conserved syntenies revealed the highest degree of similarity with the Asian and European seabass genomes (*n = *24), showing 24 pairs of one-to-one correspondences at the chromosome level (Fig. 3). For simplicity, the clownfish and four model teleosts linkage groups will be referred to hereafter as abLG (clownfish, *Amphiprion bicinctus*), lcLG (Asian seabass, *Lates calcarifer*), dlLG (European seabass, *Dicentrarchus labrax*), onLG (Nile tilapia, *Oreochromis niloticus*) and gaLG (stickleback, *Gasterosteus aculeatus*), respectively. The number of RAD loci showing significant BLAST hits to single chromosomes per clownfish linkage group (LG) ranged from 21–56 and 19–58 for Asian and European seabass respectively. For Nile tilapia (*n = *22), one-to-one homology with the great majority of clownfish abLGs was detected, with 16–49 RAD loci identified in each *Oreochromis* chromosome. Furthermore, two clownfish linkage groups (abLG3 and abLG7) mapped to a single Nile tilapia chromosome (onLG7) while clownfish abLG12 showed homology with two *Oreochromis* chromosomes (onLG17 and onLG23). The comparison with stickleback (*n = *21) revealed that three of the chromosomes shared syntenies with two clownfish linkage groups each (gaChr I with abLG9, abLG23, gaChr IV with abLG4, abLG17 and gaChr VII with abLG5, abLG24), with 7–35 RAD homologous loci being associated per clownfish abLG. A summary of the comparative analysis with the chromosomes of the four comparison species is presented in Table 2.

**Table 2:** Summary of the comparative analysis with the chromosomes of Asian Seabass (*Lates calcarifer*, lcLG), European Seabass (*Dicentrarchus labrax*, dlLG), Nile tilapia (*Oreochromis niloticu*s, onLG) and stickleback (*Gasterostes aculeatus*, gaChr).

Red Sea clownfish Map		Homologous chromosomes			
Clownfish abLG (24)	Number of loci	Asian seabass lcLG (24)	European seabass dlLG (24)	Nile tilapia onLG (22)	Stickleback gaChr (21)
1	68	12	1A	5	XVII
2	112	6	22–25	20	XII
3	71	9	19	7	XIV
4	72	8	2	2	IV
5	80	20	14	10	VII
6	37	4	10	18	III
7	75	10	6	7	XIX
8	47	13	20	12	XIII
9	46	21	13	14	I
10	57	15	16	11	XX
11	59	7_1	17	15	XVIII
12	76	17	4	17,23	VIII
13	83	19	12	19	XV
14	45	3	9	22	X
15	68	16–22	11	13	VI
16	79	11	8	4	XI
17	76	18	X	17	IV
18	65	1	15	16	XVI
19	36	23	1B	8	V
20	65	5	7	6	IX
21	54	2	5	1	II
22	60	24	18–21	9	XXI
23	29	7_2	24	23	I
24	51	14	3	3	VII

To further explore synteny, we inferred collinearity, defined as gene order within a syntenic chromosome, in all four reference species. Collinear markers indicate identical genome organization between two genomes. As expected, Asian and European seabass genomes showed the highest collinear relationships with clownfish. In both species of seabass more than half of the syntenic pairs of linkage groups (lcLGs 1, 3, 4, 5, 6, 7, 8, 13, 14, 15, 19, 20 and 22 and dlLG 1, 3, 5, 6, 7, 9, 13, 14, 15, 16, 18, 19, 20, 22 and 24, respectively) showed apparent collinear relationships with clownfish, whereas the other pairs exhibited different degrees of disrupted collinearity, suggesting intrachromosomal rearrangements (Fig. 3 and Supplementary Figs S3 and S4). Nile tilapia and stickleback genomes showed collinear relationships of linkage groups onLGs 3, 4, 5, 6, 8, 9 and 21 and gaLGs 3, 4, 8, 15, 16, 22 and 24 (Fig. 3 and Supplementary Figs S5 and S6).

To investigate loci located within protein coding sequences, mapped RAD loci were blasted against the reference clownfish transcriptome, revealing significant homology for 1937 markers −61.57% of the informative RAD loci – (Supplementary Fig. S2). Of these, 676 loci −34.9% - annotated to known proteins (Supplementary Table S5).

### Localizing molecular players potentially involved in sex change

**Figure 4:**
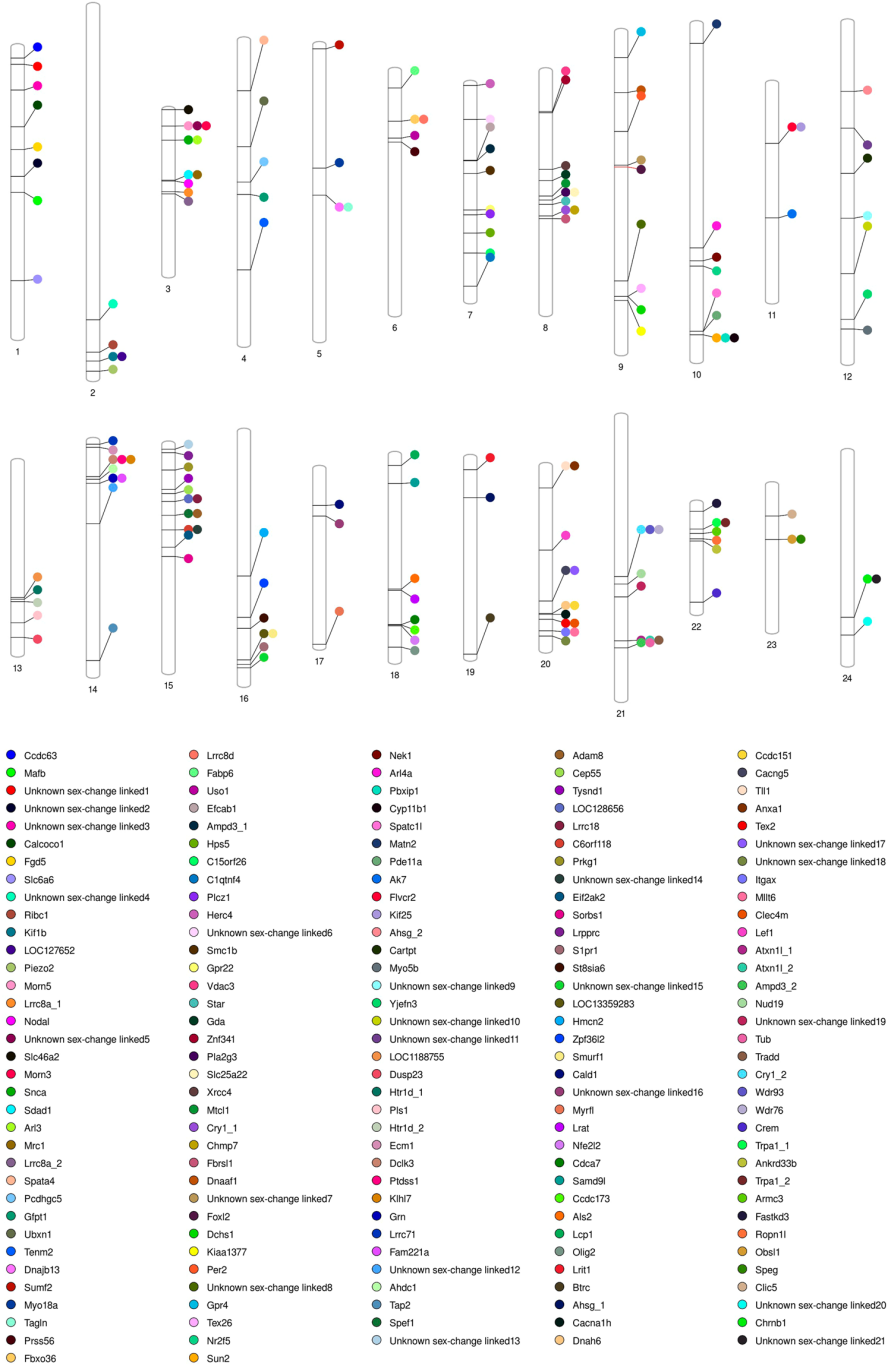
Phenogram plot showing the location of molecular players potentially involved in sex change along the clownfish linkage groups. The position of *foxl2*, proposed as a key regulator of sex change in clownfish, is highlighted in red.

The transcriptome served as well to investigate the location of mapped RAD loci showing significant similarity with transcriptome contigs encoding genes showing differential expression during sex change in clownfish. Out of 941 transcriptome contigs showing differential expression during sex change in a previous study^15^, thirteen had significant similarity with the RAD loci isolated in this study. These mapped RAD markers were scattered along abLGs 1, 4, 7, 9, 10, 13, 14, 15 and 18. Their positions are indicated in Table 3 and illustrated in Fig. 4.

**Table 3:** RAD loci potentially involved in sex change mapped onto the *A. bicinctus* linkage groups.

RAD Locus	abLG	Position (cM)	Protein ID	Protein Accession	Protein name	Protein symbol	Differential expression (Casas *et al*.^15^)
201258	1	163.675093832307	GI:657573462	XP_008290391.1	sodium-and chloride-dependent taurine transporter-like [*Stegastes partitus*]	Slc6a6	Ovary-enhanced expression
178948	4	74.3826271184075	GI:542173658	XP_005467604.1	UBX domain-containing protein 1-like isoform X1 [*Oreochromis niloticus*]	Ubxn1	Testis-enhanced expression
266196	7	25.147060130621	no significant similarity found		—	—	Testis-enhanced expression
188921	7	63.1199899241097	GI:657529007	XP_008293032.1	structural maintenance of chromosomes protein 1B-like [*Stegastes partitus*]	Smc1b	Testis-enhanced expression
268887	7	88.358913942359	GI:657554003	XP_008281445.1	probable G-protein coupled receptor 22 [*Stegastes partitus*]	Gpr22	Ovary-enhanced expression
103166	9	185.359426836091	GI:657536579	XP_008274856.1	testis-expressed sequence 26 protein [*Stegastes partitus*]	Tex26	Testis-enhanced expression
241578	10	215.31736194784	GI:657571430	XP_008289285.1	dual 3′,5′-cyclic-AMP and -GMP phosphodiesterase 11A-like [*Stegastes partitus*]	Pde11a	Testis-enhanced expression
241027	13	112.572163781802	GI:657559377	XP_008283598.1	5-hydroxytryptamine receptor 1D [*Stegastes partitus*]	Htr1d	Ovary-enhanced expression
144168	14	31.2450520202142	GI:657805406	XP_008329847.1	AT-hook DNA-binding motif-containing protein 1 [*Cynoglossus semilaevis*]	Ahdc1	Ovary-enhanced expression
143893	14	177.48764230184	GI:657593306	XP_008301218.1	antigen peptide transporter 2 [*Stegastes partitus*]	Tap2	Testis-enhanced expression
221217	15	6.96837121817013	GI:542200432	XP_003438484.2	leucine-rich PPR motif-containing protein, mitochondrial-like [*Oreochromis niloticus*]	Lrpprc	Testis-enhanced expression
60910	18	8.71205068760588	GI:657584478	XP_008296389.1	plastin-2 [*Stegastes partitus*]	Lcp1	Ovary-enhanced expression
177198	18	155.05522746894	GI:617383778	XP_007547312.1	oligodendrocyte transcription factor 2-like [*Poecilia formosa*]	Olig2	Testis-enhanced expression

Columns indicate RAD locus, clownfish linkage group (abLG), RAD locus position (cM) on the linkage group, protein identifier, protein accession, protein name and type of differential expression shown in the study by Casas *et al*.^15^.

Those transcriptome contigs that could not be assigned were screened against the four teleost genomes analyzed previously. Of the 928 transcriptome contigs in clownfish, 322 (34.7%) had a single hit to the genome of at least one of the four comparison specie genomes (131 in Asian seabass, 133 in European seabass, 164 in stickleback and 122 in tilapia). Of these, 159 (50.0%) were mapped to a putative genomic interval by collinearity-based mapping (Supplementary Table S6). These newly mapped markers encoding genes showing differential expression during sex change were located along all clownfish linkage groups, although specific regions of abLGs 3, 8, 15, 20 and 21 displayed a remarkably higher density of markers (Fig. 4).

## Discussion

Here, we present the first linkage map of *Amphiprion* using a rapid and economical method that allows constructing highly dense, coding-rich meiotic maps from the offspring of individual wild-caught parents. We applied a methodology based on the average between sexes, taking advantage of the informative markers detected for both parents, but without taking into account the differences in recombination between the two.

The constructed linkage map consisted of over 3000 polymorphic loci in the unexplored genome of the Red Sea clownfish, organized in 24 linkage groups matching the expected number of chromosomes given the haploid genome of karyotyped *Amphiprion* species. The map covered 4294.7 cM with an average of 131 loci per linkage group and an average marker distance of 1.38 cM. These data provide a good resource for future comparative genomics studies since it contains detailed information on the genomic structure of the clownfish.

The generated clownfish linkage map was compared with four reference teleost genomes to analyze the synteny and collinearity, based on presumed homologous RAD loci. We found numerous polymorphic markers that exhibited significant sequence similarity with the Asian and European seabass, Nile tilapia and stickleback genomes (Fig. 2, Supplementary Tables S3 and S4), revealing large syntenic and collinear regions within the four species (Fig. 3, Supplementary Figs S3 to S6). Our analysis revealed a one-to-one homology with the chromosomes of Asian and European seabass, the two species displaying the same chromosome number and phylogenetically more closely related. Each of the 24 clownfish abLGs exhibited strong homology with a single seabass chromosome, while collinear relationships were displayed by more than half of these syntenic pairs, for both species. For Nile tilapia, two of the clownfish linkage groups mapped to a single onLG while one of the clownfish abLGs showed homology with two *Oreochromis* chromosomes, in line with the haploid chromosome number in this species (n = 22). Both clownfish abLGs 3 and 7 showed homology to *Oreochromis* linkage group 7, a known fused chromosome in an event that took place before the divergence of the main East African cichlid groups^34^. Similar results were reported in common Pandora^13^ and red drum^35^.

Similarly, six of the clownfish linkage groups merged into three chromosomes in stickleback exhibiting homology in a pair-wise manner, consistent with the haploid chromosome number in *G. aculeatus* (n = 21). This has been reported in previous studies and reflects three simple pairwise fusions of six ancestral chromosomes in the stickleback genome^11^. In our study, stickleback chromosomes I, IV and VII are a fusion of clownfish abLG9 and abLG23, abLG4 and abLG17 and abLG5 and abLG24, respectively. In addition, each portion of these three stickleback chromosomes is in general orthologous to a single clownfish abLG. The first 5 Mb of stickleback gaChr I has orthologs on clownfish abLG9, while the last 10 Mb contains orthologs of genes on abLG23 (Fig. 3). For gaChr IV, the first 17.8 Mb and the last 14.8 Mb have orthologs distributed broadly over abLG4 and abLG17, respectively. Finally, genes located on the first 10 Mb of gaChr VII have clownfish orthologs distributed along abLG24 and the last 15 Mb segment contains orthologs on abLG5. The same three stickleback chromosomes share synteny with a pair of linkage groups in *Xiphophorus maculatus*^11^, Asian seabass^14^ and *Pagellus erythrinus*^13^. Nile tilapia and stickleback genomes showed collinear relationships of seven syntenic pairs each. Thus, gene order within a syntenic linkage group was substantially retained between clownfish and both species of seabass, while a lower degree of collinearity was found between *Amphiprion* and *Oreochromis* and stickleback, where a number of interchromosomal rearrangements appear to have occurred. Overall, the extensive synteny and chromosome homology of clownfish with all four species suggest a relatively conserved genomic structure among the groups and provide evidence of the robustness of the produced linkage map.

To further validate the map, we also conducted a BLAST search against the reference clownfish transcriptome revealing a remarkably high percentage −63.98% - of the RAD loci showing significant homology. Of these 34.9% - annotated to known proteins. This relatively high percentage – more than one third - of the RAD loci identified as being homologous to coding regions could be due, at least in part, to the RAD strategy employed, where a restriction enzyme with GC-rich recognition sites was used in library construction. An enrichment analysis of RADseq loci that mapped to protein functions reported a significant enrichment of genomic regions encoding proteins with Methionine-Leucine (ML) amino acid stretches - translated sequence of the *SphI* recognition site - when using this restriction enzyme^36^.

Finally, the high-density linkage map generated in our study provided a platform for mapping of genes showing differential expression during sex change in *A. bicinctus*. Up to date, very little information exists regarding the molecular mechanism underlying sex change and gonadal restructuring in hermaphrodites. In clownfish, a previous study^15^ revealed close to 1000 transcriptome transcripts showing differential expression during sex change. Their expression patterns and regulatory mechanisms of those with annotation in public databases indicated that they are part of a signaling network responsible for the development of sex specific gonads. In the present study, we analyzed them using a two-step strategy. First, the set of transcripts was blasted against the set of RAD loci isolated in this study, revealing 13 genes showing differential expression during sex change that showed significant similarity (Table 3). They were found to be scattered along the clownfish linkage groups abLGs 1, 4, 7, 9, 10, 13, 14, 15 and 18. Next, we used a collinearity-based mapping approach to assign putative locations of the clownfish genes differentially expressed during sex change previously unmapped. Using the information from the genomes of Asian and European seabass, Nile tilapia and stickleback we inferred the likely positions of additional 159 transcripts potentially involved in sex change, showing the validity of this approach in localizing candidate genes in non-model species for which a reference genome is unavailable. The transcripts were distributed along all abLGs, although the number of sex change linked genes was highly variable among linkage groups. These results indicate that multiple regions seem to be associated with sex change in clownfish, highlighting a complex genetic architecture with candidate genes showing differential expression during sex change scattered throughout all linkage groups. Linkage groups abLG4, abLG11, abLG17, abLG19, abLG23 and abLG24 showed the lowest density of sex change linked genes -3 - per linkage group while abLG3, abLG8, abLG15, abLG20 and abLG21 displayed a remarkably higher density with 11 to 13 markers per linkage group. Several testis associated proteins - spermatogenesis-associated protein 4 (Spata4), testis-expressed sequence 26 (Tex26), sperm flagellar protein 1 (Spef1), testis-expressed sequence 2 (Tex2) - were allocated to linkage groups abLG4, abLG9, abLG15 and abLG20 respectively. Moreover, the forkhead box L2 (*foxl2*), a transcription factor known to play a a decisive role in the ovarian differentiation process in various vertebrate species, including fishes^37^ was located on abLG9 (Fig. 4). Foxl2 is involved in the regulation of estrogen synthesis via direct modulation of aromatase expression and possibly the entire steroidogenic pathway^38^ and also required to maintain the identity of ovarian granulosa cells^39^. In *A.bicinctus*, it has been shown that the sex steroidogenic machinery plays a central role during sex change with *foxl2* acting as a main transcriptional regulator in the activation of the female pathway driving the gonadal transformation from testis to ovary^15^. Foxl2 also regulates the promoter activity of the steroidogenic acute regulatory gene (*star*) which controls the rate-limiting step in steroid hormone synthesis and is required for normal ovarian steroid production in mice and humans^40,41^. Star was mapped to abLG8 while the gene encoding steroid-11β-hydroxylase (*cyp11b1*), another steroidogenic enzyme involved in gonadal differentiation in fish^42^, was located on abLG10.

The present study constitutes the most complete analysis exploring the location of loci encoding genes potentially involved in the genetic mechanism governing social sex change and gonadal restructuring in a protandrous hermaphrodite to date.

## Methods

### Ethics statement

This research was carried out under the general auspices of King Abdullah University of Science and Technology’s (KAUST) arrangements for marine research with the Saudi Arabian Coast Guard and the Presidency of Meteorology and Environment. The animal use protocol was approved by KAUST’s Biosafety and Ethics Committee (KAUST does not provide specific approval number). The methods in this research were carried out in accordance with the approved guidelines.

### Mapping family

A mating pair of adult specimens guarding a clutch of eggs was located on SCUBA off the Saudi coast of the Red Sea. The eggs were carefully detached using a knife and collected in a plastic bag. The male and female parents were left on site after their fin clips were sampled. Fin clips were preserved in 99% ethanol and kept at −20° until DNA extraction. Eggs were transported to the laboratory and transferred to a small aquarium containing aerated and filtered sea water. Six hours later, eggs started hatching and each larva was preserved in an Eppendorf tube filled with 99% ethanol.

We used massively parallel DNA sequencing to develop a linkage map by genotyping F1 offspring of a single female and male clownfish (*Amphiprion bicinctus*). A total of 94 full-sib F1 progeny were sampled and used, together with the parents, for the RAD library construction (96 fish in total).

### RAD library construction and sequencing

Genomic DNA of parents and their F_1_ progeny was extracted from the preserved samples using a DNeasy Blood & Tissue Kit (Qiagen, Valencia, CA). Quality and concentration of genomic DNA were checked using a Bioanalyzer 2100 (Agilent, Santa Clara, CA) and a Qubit fluorometer (Invitrogen, Carlsbad, CA) prior to library creation.

Approximately 1 μg of purified DNA per sample was processed to obtain two RAD libraries each including 48 individuals. Genomic DNA from each individual was digested at 37 °C for 15 minutes with the restriction endonuclease *SphI* (GCATG|C recognition site) (NEB, Ipswich, MA). Modified Illumina adapters containing five nucleotides of barcode sequence unique (P1 adapters) to an individual in the library were ligated with T4 DNA ligase (NEB, Ipswich, MA) to allow sample multiplexing. Ligation reactions were cleaned using the Agentcourt AMPure XP system (Beckman Coulter, Brea, CA) followed by two rounds of purification in 80% ethanol. Each sample was quantified again using a Qubit fluorometer (Invitrogen, Carlsbad, CA) prior to sample multiplexing. The pooled samples were then sheared using a Covaris S-Series ultrasonicator (Covaris, Woburn, MA) and size-selected to isolate DNA fragments spanning 300–500 bp by agarose gel electrophoresis. The Zymoclean Large Fragment DNA Recovery Kit (Zymo Research, Irvine, CA) was used to purify and recover the DNA from the gel following manufacturer´s instructions. The NEBNext Ultra DNA II Library Prep Kit (Illumina, San Diego, CA) was then used for End Repair and dA-Tailing, followed by the P2 Adaptor Ligation step to enable selective PCR. The reactions were then purified with a QIAquick column (Qiagen, Valencia, CA).

The samples were amplified using the NEBNext DNA Library Prep Master Mix Set by 10 cycles of PCR following a scaled-down version of the manufacturer’s protocol. Next, the samples were purified by agarose gel electrophoresis followed by cleaning with the Zymoclean Large Fragment DNA Recovery Kit (Zymo Research, Irvine, CA) to obtain the sequencing libraries. The obtained RAD libraries were sequenced on four lanes on a HiSeq. 2000 platform (Illumina, San Diego, CA) in 100-bp paired reads, at the KAUST Bioscience Core Laboratory (Thuwal, Saudi Arabia).

### Genotyping

Raw reads were analyzed in Stacks 1.21^43^. Using the “process_radtags” pipeline, raw reads for each individual in the RAD tag library were demultiplexed and trimmed to a common length of 95 bp in FASTQ format. Those reads with an average Phred score <20 (in a 5 bp sliding window) were discarded. Retained reads from each sample were analyzed for building loci and calling SNPs *de novo* using the “denovo_map.pl” pipeline following the genetic map guidelines. The parameters used in Stacks were: minimum number of reads required to form a stack (m) was set to 8; maximum number of mismatches between loci for individual (M) was set to 2; maximum number of mismatches when aligning secondary reads to primary stacks (N) was set to 3; maximum number of mismatches between loci when creating a catalog (n) was set to 2. Genotypes were exported to a file in onemap format. We then filtered this file and kept only those loci that: 1) followed Mendelian segregation assuming codominance (p > 0.5, Chi-square test) and 2) were present in at least 80% of the offspring. This final “RAD loci” data set consists of 95 bp sequences. To extend their length, the associated paired-end reads were retrieved and assembled obtaining contigs that span several hundred bases of flanking sequence of randomly sheared genomic fragments anchored at the same restriction site (referred to as “RAD contigs” hereafter). Briefly, we used the “sort_read_pairs.pl” program implemented in Stacks to collate the paired-end sequences for each catalog locus in the final dataset. We then used Velvet version 1.2.10^44^ to assemble contigs for each locus using the following parameter set: -H 17, -M 150, which maximized the number and length of assembled loci.

### Linkage map construction

We used the R package “onemap”^45^ to build the genetic map as follows. Briefly, genotypes of the 94 offspring were coded using the notation described in^46^ using the program “genotypes” implemented in Stacks. Genotypes were read into onemap using the “read.outcross” function. We estimated two point recombination fractions among all pairs of markers using two-point tests (rf.2pts function) with LOD scores set to 4 and the maximum recombination fraction set to 0.4. Markers were then assigned to linkage groups using the “group” function. Different LOD and max recombination fractions were tested for grouping. A LOD score of 14 and max recombination fraction of 0.4 was selected based on the number of linkage groups yielded -24- which coincides with the number of chromosome pairs in *Amphiprion bicinctus* (2n = 48)^47^ and the minimization of the number of unassigned markers. Once all 24 linkage groups were defined, genetic mapping for each group was performed using the Kosambi mapping function to convert recombination fractions into centiMorgan and the “order.seq” function with an initial number of markers to compare of 6 and a LOD threshold of 3 to find the best order among markers. This function automates a procedure were initially a set of the most informative markers is analyzed and ordered, and then each of the remaining markers is added one at a time at the location with the highest likelihood. Thus, markers that were not uniquely mapped were assigned to the most likely position. Finally, we used the function “ripple.seq” using predefined parameters to check for alternative orders of markers (given that an exhaustive search is not performed in the previous step).

### Comparative genomics

The assembled RAD contigs associated to the mapped RAD loci in the Red Sea clownfish were used in a comparative analysis with the genomes from the following reference teleosts: European seabass (*Dicentrarchus labrax*, dicLab v1.0c http://seabass.mpipz.de)^48^, Asian seabass (*Lates calcarifer*, http://seabass.sanbi.ac.za/Lates_calcarifer/)^49^, Nile tilapia (*Oreochromis niloticus*, O_niloticus_UMD1)^50^ and stickleback (*Gasterosteus aculeatus*, Ensembl 88)^51^. BLASTN (BLAST+ version 2.2.26) searches with an *e*-value cutoff of 10^−9^ were conducted.

The annotation of each sequence that aligned on the clownfish map was manually verified. To eliminate repetitive sequences, loci with more than 10 hits were excluded; for the remaining loci the top hit per sequence was retained and considered homologous to the RAD locus. The chromosomal locations and linkage groups (LGs) of RAD loci were recorded. Putative conserved syntenies were identified when most loci of a clownfish LG (abLG) were homologous to loci located on a single chromosome in the reference species.

The remaining unmatched RAD loci were aligned to the recently published reference clownfish transcriptome assembly (GenBank Assembly GDCV00000000.1; https://www.ncbi.nlm.nih.gov/nuccore/GDCV00000000) allowing one best hit per marker with an e-value cut-off of 10^−20^. This strategy provided anchors for positioning loci that did not directly match the reference genomes. A Fasta file with marker sequences replaced by assigned transcripts (if applicable) was obtained and used for comparative mapping by BLAST to each of the four reference genomes, with the same parameters described above.

Circos software^52^ was used to represent syntenies. The distances among genes on the same chromosome of Asian seabass, European seabass, Nile tilapia and stickleback are given in base pairs, whereas the distances among markers on the linkage groups of clownfish are given in centiMorgans. The conservation of the order of orthologous loci within linkage groups/chromosomes - collinearity - between the clownfish and the four reference species was visualized using Oxford grids^53^. The positions plotted are proportional to physical length (x-axis) and Kosambi cM (y-axis).

To investigate loci located within protein coding sequences, assembled RAD contigs were blasted against the recently published reference clownfish transcriptome (GenBank Assembly GDCV00000000.1; https://www.ncbi.nlm.nih.gov/nuccore/GDCV00000000) using an e-value cut-off of 10^−20^.

### Mapping of molecular players potentially involved in sex change

A published dataset of genes showing differential expression during sex change in a recent transcriptomic study^15^ was retrieved and tested in order to investigate their genomic position on the consensus clownfish linkage map. Their sequences were used in BLAST searches against the set of assembled RAD contigs with an e-value threshold of 10^−20^. Those that could not be assigned were re-analyzed following a strategy based on shared collinearity with well-characterized genomes which allows inferring positions of unmapped candidate genes of interest in non-model species^35,54^. Briefly, cDNA sequences of gene transcripts not assigned in the previous step were screened against the genome of each of the four comparison species, using BLAST+, as above. Only transcript sequences with single hits to the genome of at least one comparison species were retained. For each locus, it was determined whether the locus was located in a region of shared collinearity, defined as regions consisting of at least two loci that share a common marker order between the clownfish linkage map and the genome of a comparison species, and which are uninterrupted by other mapped loci. Ordering mismatches between markers separated by less than five percent of the total length of a linkage group/chromosome were allowed in order to maximize detection of informative syntenies otherwise disrupted by local rearrangements or small errors in genome assembly and map order. When transcript sequences were located in a region of shared collinearity the nearest left and right flanking loci and their positions were recorded. Information from all four species was then used to infer the specific location in the clownfish linkage map and the smallest possible interval within the region into which the locus could be positioned. The location of these genes was visualized using PhenoGram software^55^.

### Availability of supporting data

The raw RAD sequence data set used in this study was submitted to the Sequence Read Archive under accession number PRJNA399257.

## Electronic supplementary material

Supplementary Information

Supplementary Tables S1 to S6
