# Supplementary material for: High-Throughput Sequencing and Linkage Mapping of a Clownfish Genome Provide Insights on the Distribution of Molecular Players Involved in Sex Change
Source: Sci Rep. 2018 Mar 6;8:4073. doi: 10.1038/s41598-018-22282-0 (PMC5840384; doi:10.1038/s41598-018-22282-0)
Supplement: Supplementary file 1 — Supplementary Information [file 41598_2018_22282_MOESM1_ESM.doc]

**High-Throughput Sequencing and Linkage Mapping of a Clownfish Genome Provide Insights on the Distribution of Molecular Players Involved in Sex Change**

**Laura Casas**^1,2,*^**, Pablo Saenz-Agudelo**^1,3^**, Xabier Irigoien**^1,4,5^

^1^ King Abdullah University of Science and Technology (KAUST), Division of Biological and Environmental Science & Engineering, Red Sea Research Center, Thuwal 23955-6900, Saudi Arabia

^2^ Present address: Institute of Marine Research (IIM-CSIC), 36208 Vigo, Spain

^3^ Present address: Instituto de Ciencias Ambientales y Evolutivas, Universidad Austral de Chile, Valdivia 5090000, Chile

^4^ Present address: AZTI - Marine Research, Herrera Kaia, Portualdea z/g – 20110 Pasaia (Gipuzkoa), Spain.

^5^ Present address: IKERBASQUE, Basque Foundation for Science, Bilbao, Spain.

* Corresponding author: Laura Casas [lalaucas@gmail.com](mailto:lalaucas@gmail.com)

**Supplementary data:**

Includes an additional file with Tables S1 to Table S6 in excel format (S1_S6_Tables.xlsx) and six supplementary figures (Supplementary Figure S1 to S6).

Table S1. Number of sequencing reads assigned to each individual after demultiplexing and length filtering and Stacks summary data: number of reads used in Stacks, number of unique Stacks, average depth of coverage, number of loci matching catalog, percentage of loci matching catalog (%), number of polymorphic loci, number of loci with mendelian segregation (p>0.05), number of segregating type consistent with outcross.

Table S2. Detailed information of the RAD loci used for the linkage mapping: RAD locus name, clownfish linkage group (abLG) assigned, position in the abLG in centiMorgans, RAD locus sequence, RAD contig sequence.

Table S3. Table S3. Comparative genomic information of the RAD loci used for linkage mapping: RAD locus, clownfish linkage group, position in centiMorgans, linkage group in the comparison species, start of alignment, end of alignment, percentage of identity, e-value reported by Blastn; comparison species -the species in which a syntenic relationship with clownfish was detected: 1: Asian seabass (Lates calcarifer), 2: European seabass (*Dicentrarchus labrax*), 3: Nile tilapia (Oreochromis niloticus), 4: three-spined stickleback (*Gasterosteus aculeatus*).

Table S4. Table S4. Comparative genomic information of the transcriptome-anchored RAD loci used for linkage mapping: RAD locus, assigned transcriptome contig, clownfish linkage group, position in centiMorgans, linkage group in the comparison species, start of alignment, end of alignment, percentage of identity, e-value reported by Blastn; comparison species -the species in which a syntenic relationship with clownfish was detected: 1: Asian seabass (*Lates calcarifer*), 2: European seabass (*Dicentrarchus labrax*), 3: Nile tilapia (*Oreochromis niloticus*), 4: three-spined stickleback (*Gasterosteus aculeatus*); assigned transcriptome contig sequence.

Table S5. Annotation of the RAD loci used for the linkage mapping against the clownfish reference transcriptome.

Table S6. Summary of synteny-mapped loci: sex-transcript ID, consensus clownfish linkage group - defined as the abLG to which the locus was mapped, consensus position range in centiMorgans, protein ID, protein name, flanking loci - defined as the closest clownfish markers between which the sex-transcript could be mapped based on synteny with one or more comparison species; comparison species -the species in which a syntenic relationship with clownfish was detected: 1: Asian seabass (*Lates calcarifer*), 2: European seabass (*Dicentrarchus labrax*), 3: Nile tilapia (*Oreochromis niloticus*), 4: three-spined stickleback (*Gasterosteus aculeatus*).

**
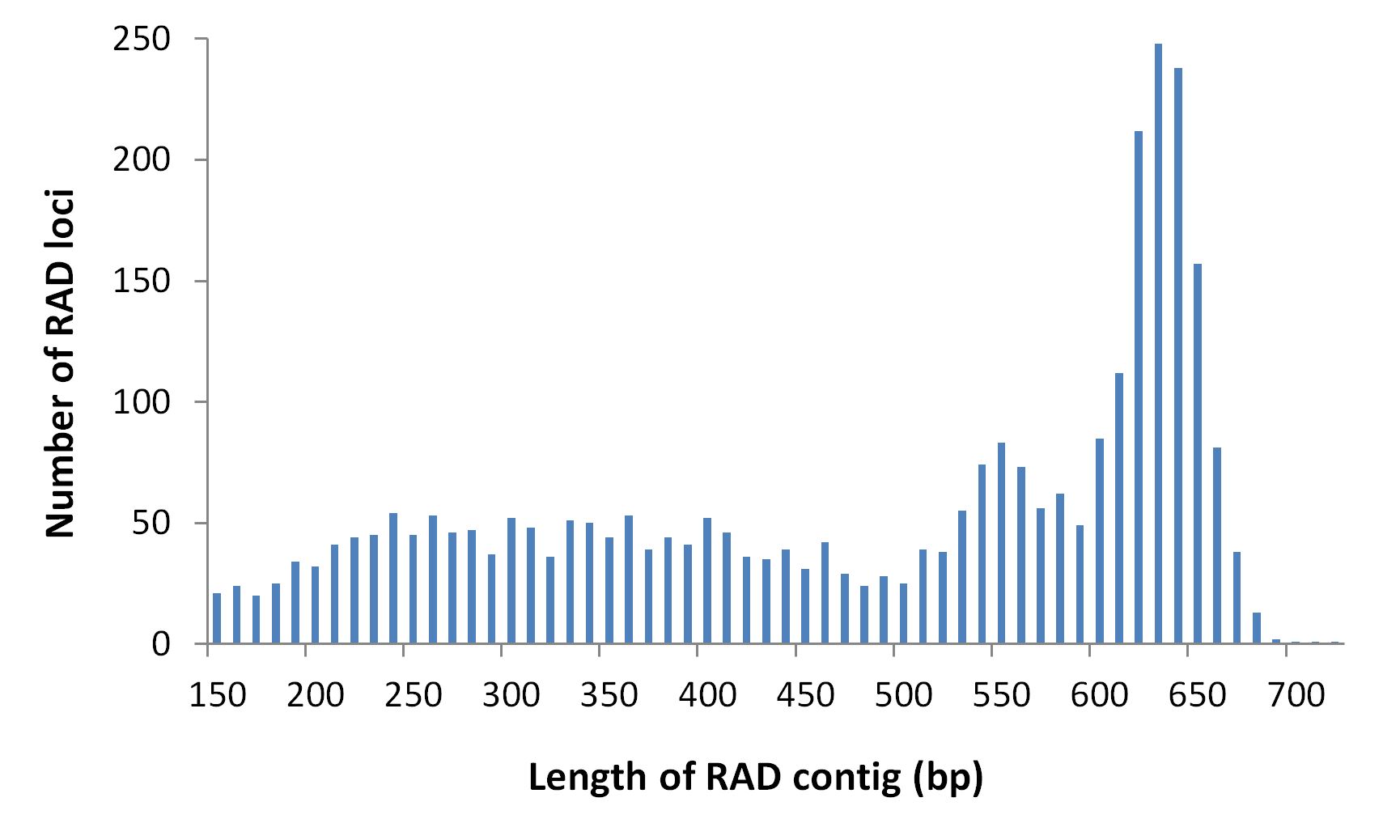
**

**Supplementary Figure S1.-** Size distribution of RAD assembled paired-end contigs. The x-axis and y-axis are respectively the contig length in base pairs and the number of RAD contigs with each length.


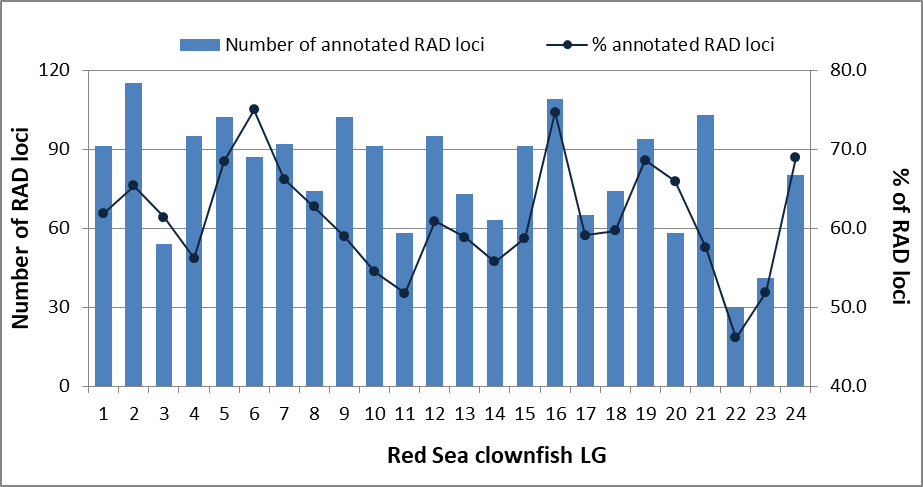


**Supplementary Figure S2.-** Number and percentage of annotated RAD loci in each linkage group (LG) of the Red Sea clownfish, located in within protein coding sequences.


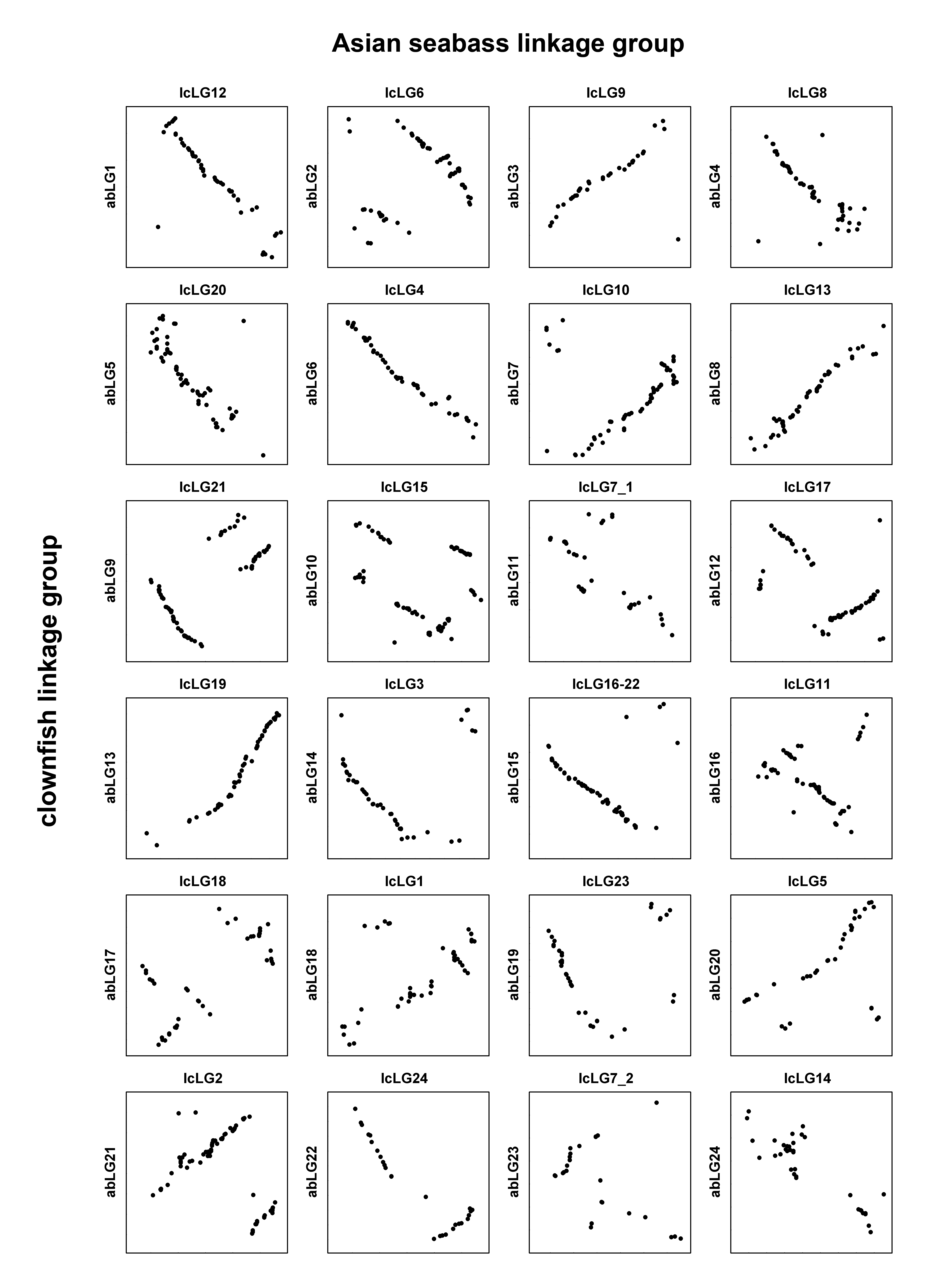


**Supplementary Figure S3.-** Oxford grids displaying the collinearity of the syntenic chromosomes of the Red Sea clownfish and Asian seabass genomes. Each dot represents the position of a homologous locus. The x-axis is proportional to physical length while the y-axis is proportional to Kosambi cM.


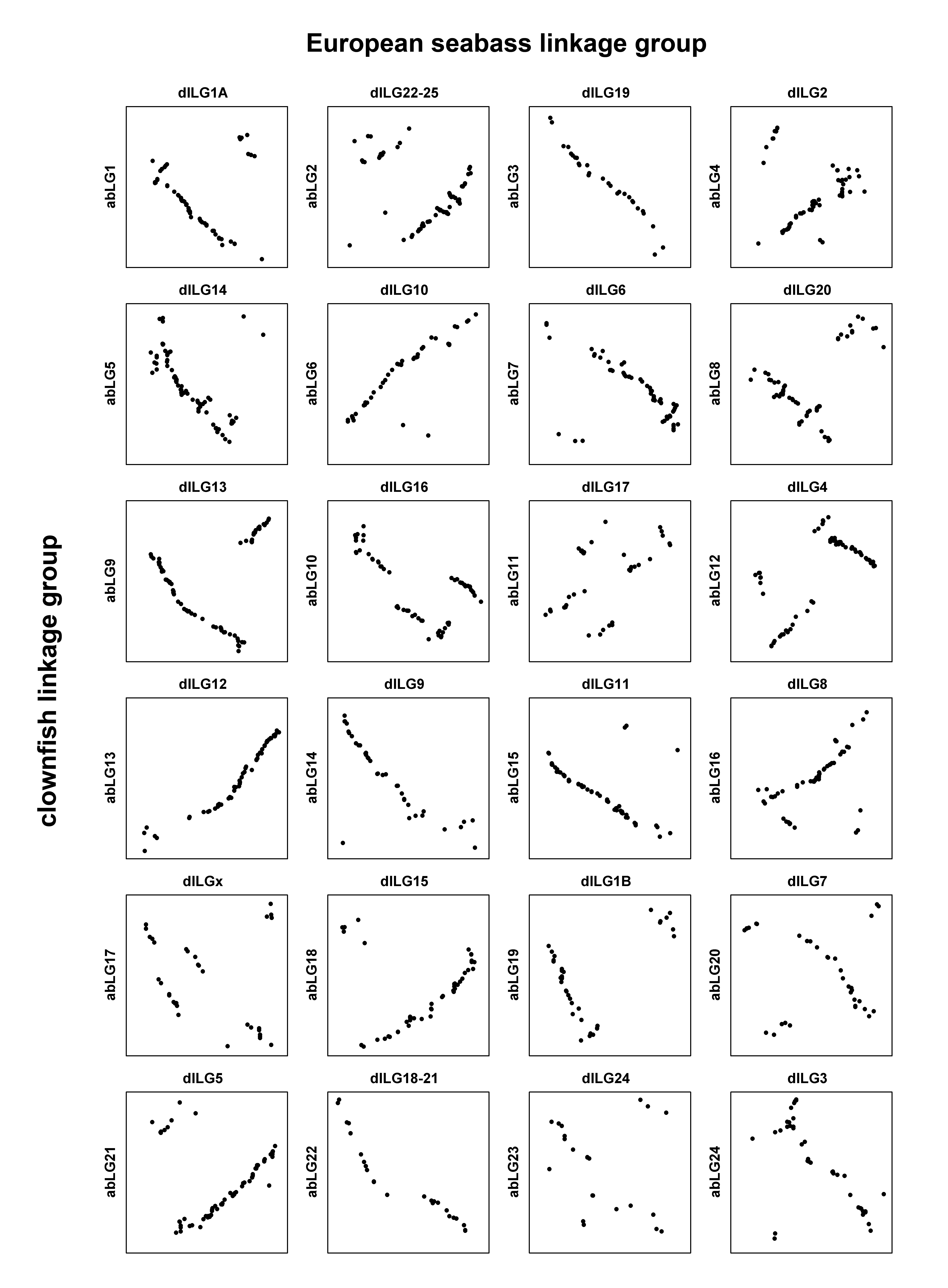


**Supplementary Figure S4.-** Oxford grids displaying the collinearity of the syntenic chromosomes of the Red Sea clownfish and European seabass genomes. Each dot represents the position of a homologous locus. The x-axis is proportional to physical length while the y-axis is proportional to Kosambi cM.


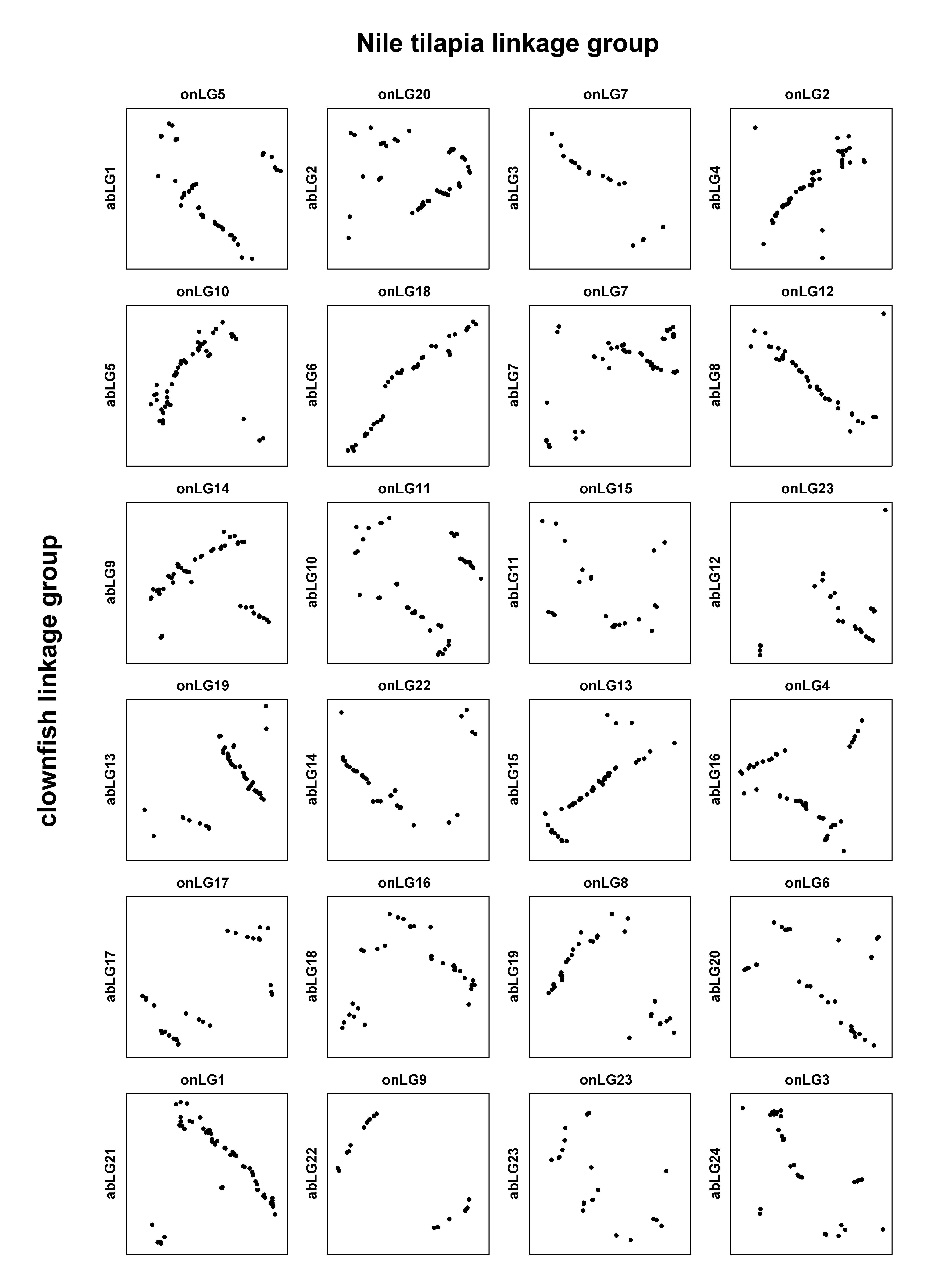


**Supplementary Figure S5.-** Oxford grids displaying the collinearity of the syntenic chromosomes of the Red Sea clownfish and Nile tilapia genomes. Each dot represents the position of a homologous locus. The x-axis is proportional to physical length while the y-axis is proportional to Kosambi cM.


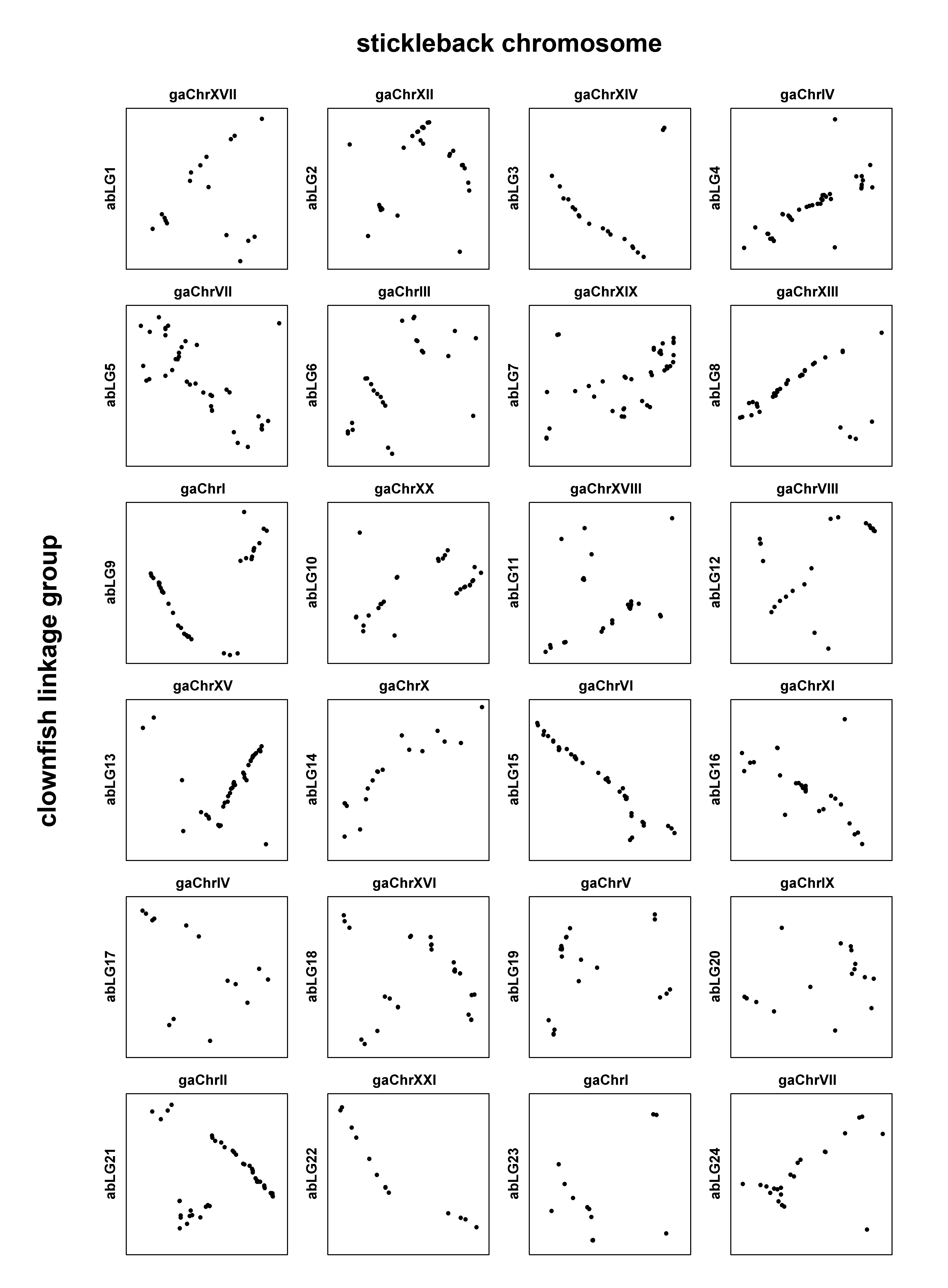


**Supplementary Figure S6.-** Oxford grids displaying the collinearity of the syntenic chromosomes of the Red Sea clownfish and stickleback genomes. Each dot represents the position of a homologous locus. The x-axis is proportional to physical length while the y-axis is proportional to Kosambi cM.
